# Secure Route-Obfuscation Mechanism with Information-Theoretic Security for Internet of Things

**DOI:** 10.3390/s20154221

**Published:** 2020-07-29

**Authors:** Abid Rauf, Zhaohong Wang, Hasan Sajid, Muhammad Ali Tahir

**Affiliations:** 1 National University of Sciences and Technology (NUST), School of Electrical Engineering and Computer Science, Islamabad 44000, Pakistan; ali.tahir@seecs.edu.pk; 2 Department of Electrical and Computer Engineering, California State University, Chico, CA 90802, USA; zwang25@csuchico.edu; 3 National University of Sciences and Technology (NUST), School of Mechanical and Manufacturing Engineering, Islamabad 44000, Pakistan; hasan.sajid@smme.nust.edu.pk

**Keywords:** information-theoretic security, multipath routing, Internet of things

## Abstract

As accessibility of networked devices becomes more and more ubiquitous, groundbreaking applications of the Internet of Things (IoT) find their place in many aspects of our society. The exploitation of these devices is the main reason for the cyberattacks in IoT networks. Security design is still an open problem and a crucial step in making IoT applications successful. In dicey environments, such as e-health, smart grid, and smart cities, real-time commands must reach the end devices in the scale of milliseconds. Traditional public-key cryptosystem, albeit necessary in the context of general Internet security, falls short in establishing new session keys in the scale of milliseconds for critical messages. In this paper, a systematic perspective for securing IoT communication, specifically satisfying the real-time constraint against certain adversaries in realistic settings. First, at the network layer, we propose a secret random route computation scheme using the software-defined network (SDN) based on a capability scheme using the network actions. The computed routes are random in the eyes of the eavesdropper. Second, at the application layer, the source breaks command messages into secret shares and sends them through the network to the destination. Only the legitimate destination device can reconstruct the command. The secret sharing scheme is efficient compared to PKI and comes with information-theoretic security against adversaries. Our proof formalizes the notion of security of the proposed scheme, and our simulations validate our design.

## 1. Introduction

The fast expansion of network-enabled devices makes our lives more convenient than ever. From smart homes to smart cities, an upgrade of network-connected devices is everywhere. Thus, an era of the IoT has come. Some IoT applications are time-critical. For example, in critical infrastructures such as the smart grid, the utility should deliver controlling commands to end-user devices at the scale of milliseconds [1]. In IoT surveillance systems, the detection of intruders should also trigger the alert in milliseconds. Mainly, concerns are raised over the security of the IoT [2].

IoT devices can range from medical devices, smart home sensors, autonomous vehicles as well as neuclear technology. Due to the light nature of IoT, it is vulnerable to cyber-attacks as communication is mostly done without confidentiality and authenticity.

An essential aspect of the success of IoT applications is information security. The receiver node needs to authenticate the sender node, and the messages themselves should be appropriately encrypted against illegitimate adversaries. In the context of general internet communication, the public key infrastructure (PKI) has become the de facto security mechanism for authentication and encryption. However, it is not always a good fit for PKI in IoT due to its light nature—computing resources and energy storage. First, nodes in IoT usually have limited computing resources compared to general-purpose computers. Running PKI algorithms will deplete the computing resources as well as battery energy. Second, the encryption of public-key cryptosystems expands the size of messages to thousands of times. Consequently, much-expanded ciphertext increases the use of bandwidth. In turn, the inefficiency in the communication and decryption makes PKI so slow that it cannot satisfy real-time requirements of critical IoT applications [1].

With the advent of quantum computers, mathematically hard problems that are almost impossible for classical digital computers become much easier. Mathematical hard problem based cryptographic primitives such as the PKI schemes, are vulnerable to computing machines with high power [3]. With the rise of quantum computing platforms, the continuous usage of conventional Public key primitives established hundreds of years ago, has opened doors for attackers to crack the implementation [4]. A classical encryption can exponentially decrypted using the quantum computers [4].

Considering the ever more critical IoT applications in the critical domains with time constraints on message passing, we propose a secure message delivery framework with information-theoretic security in this paper. Unlike the PKI framework, we endeavor to guarantee real-time message passing for time-critical scenarios and to relieve the assumption on the computational power of adversaries.

Latest advancements in the field of computer networks has introduced a novel generation of software-defined network (SDN), known as the “SDN Controller”, which permits to manipulate the general performance of the network [5].

The facilitation of strategies by the controller such as, responses to security threats, fine-grained traffic filters, and effective depolyment of security policies on the fly. Our proposed framework makes use of SDN to mitigate the vulnerabilities. The controller is central and has visibility over its domain. Controllers can manage the security of the IoT architecture.

**Figure 1:**
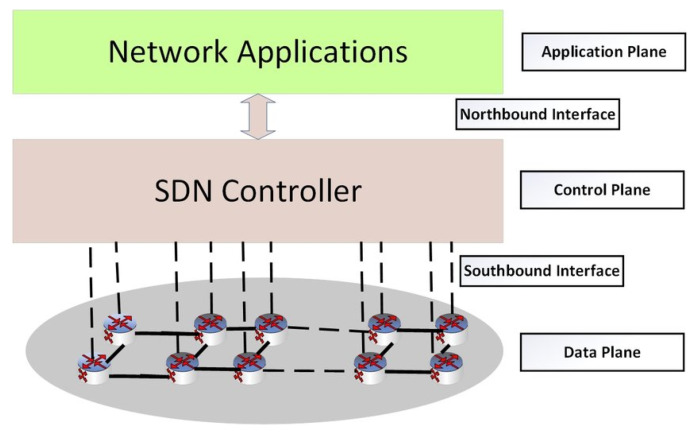
Software-defined network (SDN) architecture.

In Figure 1 shows SDN architecture and highlight security vulnerabilities. The SDN controller is the key part of the control plane, and it is responsible to develop interfaces to other SDN planes. The SDN controller interconnect Application Programming Interface (API) with application plane through Northbound Interface and enables the communication between switch and controller through a Southbound interface in data plane. Open Networking Foundation maintained the essential protocol, i.e., OpenFlow used the Southbound interface. Use of SDN technology in IoT has open the door of deployment of services by decoupling of business and data planes. Due to the fully distributed nature of the control, new services like IoT in a fully optimized manner are deployed.

Due to mixture of various components in the SDN architecture, and communication between these components, not only the efficiency and reliability, security is a very important aspect. Malicious intruders attack the weaknesses, by means of taking control of the controller in an SDN network. The change of routes by changing the flow table entries of devices leads to the Man in the Middle Attack (MITM).

With all the above observations, our unique contribution in this domain is as follows. Firstly, we have proposed a framework with SDN controlling secret shares is the first novel framework utilizing secret schemes for traffic obfuscation. We make the routing paths random from time to time employing SDN control. Consequently, the routes become unpredictable in the eyes of an eavesdropper. Intuitively, the routes look random to the eavesdropper—the security relates to randomly guessing the ever-changing correct route out of a large set of routes. Second, we turn to information-theoretic secure encryption schemes, in contrast to the public key scheme which is not suitable for real-time applications. Therefore, intercepting shares less than the threshold will never reveal the original message. The formal proof is summarized in the paper. Third, using the well-established Shamir’s secret sharing scheme, even if a share is intercepted and altered by an adversary, the receiver node will detect the alteration at the reconstruction stage. Furthermore, we proposed a node capability-based mechanism that supports the routing algorithm in maintaining efficiency. Our framework comes with security proof and simulation to validate our design.

Next, we formulate our design problem as well as the adversary model.

### Problem Statement and Adversarial Modeling

We consider IoT scenarios that are organized as IP networks. Any end-users are associated with end node devices, i.e., the IoT devices. The communication between nodes is through routers and gateways. Refer to Figure 1, the routers, and gateways form the backbone of the IoT network. The communication in the IoT scenario happens as follows. The administrator or an end node may be sending real-time controlling commands to another node. The end node will find one of the nearby routers to relay its message. The routers in between form necessary routes to further deliver the message to its destination. The SDN controller is responsible for figuring out possible routes for the messages to pass through routers.

While the scenario is general IoT, we focus on information-theoretic security against adversaries modeled as follows. We assume that an adversary may intrude into one of the routing paths in the IoT scenario. They may passively spy on the messages sent through the communication lines and may attempt active assaults such as the replay assaults and DoS assaults, and they may actively tamper encrypted communication. The adversary mainly tries to decrypt the messages, and to deduce the network’s process by finding the opportunities of launching sophisticated attacks.

The overall idea of our proposed framework is to obfuscate traffic patterns to the adversary and encrypting messages, so the adversary cannot decipher the message if the adversary only has access to a few routing paths. We do no longer assume the adversaries’ computational capability. The reason is that adversaries with quantum computing power should be taken into consideration in the design of securing light weighted IoT devices.

In Section 2 we have reviewed all the important aspects related to our design. Section 3 describes our detailed proposed framework. Section 4 provides security proof for our framework. Section 5 presents the simulations that validate our design. Finally, Section 6 concludes the paper.

## 2. Related Work

Our framework will utilize several primitive building blocks. The first one is the SDN controlled routing. The area of routing in SDN has been aggressively explored. The SDN controller can collect link states from SDN-enabled routers to form a complete interpretation of the network. [6]. Therefore, the SDN controller helps to compute the shortest routing paths for nodes in the network [7]. The SDN could be deployed to secure the entire network because it has the role of resource allocation, especially the placement of secure-related middleboxes [8].

A work that is particularly relevant to our work is the routing paths construction in SDN. To address the issue of SDN routing, several studies has been carried out. Shin et al. proposed four basic routing algorithms to maximize the security in utilizing the resources in SDN and the idea of Network Services Virtualization (NSV) [9]. Shen et al. proposed a data structure to abate the tree and cost of recovery [10]. A unique cost prototype, which simulates the usage expenses, hyperlink resources, and nodes, to maximize the throughput in the network by Huang et al. [11]. As compared to them, we have focused more on the security using obfuscation of routing. Furthermore, the information-theoretic proof authenticates that the scheme is secure against the adversaries with unlimited power.

A segment routing algorithm is proposed by Lee and Sheu [12] with segment routing, which focuses on balancing the traffic load on different routes and reducing the size of the network packet. Lee et al [13] proposed a routing aware algorithm. Huang et al. [14] and Wan et al. [15] proposed an assessment system that focused on SDN routing. SDN based routing called RouteGuardian, was proposed by Wang et al. [16], which considered the competences of the SDN switch nodes. RouteGuardian supports dynamic routing, but the efficiency of the proposed scheme has high time complexity.

There are various proposals suggesting to change network configurations dynamically to mislead the attacker. Previously more focus was on changing the network architecture to obscure traffic [17], applying cryptographic primitives on packets, and randomly changing the IP addresses and TCP/UDP ports. The attack surface of SDN was discovered by Yoon et al. [18]. As compared to the work done in past, our work’s prime focus is security and obfuscation based upon random routing path construction and information-theoretic security.

PKI is a mechanism developed involving the trust of third party mediation. High computation cost near to exponentiation is required to generate and verify the signature. Fouda et al. [19] proposed Diffie–Hellman along with a hashing-based authentication. In [20] among smart meters, a cryptographic identity based mutual authentication mechanism by Nicanfar et al. As, all these proposed schemes make use of PKI, they require exponential computational cost. In the potential threat of quantum computer adversary, all number-theoretic-based public key cryptosystems will lose their security. Researchers have worked to find alternative public-key primitives, such as lattice-based cryptography and code-based cryptography [21,22,23,24].

Alternatively, people use information-theoretic secure schemes to secure messages. In fact, messages are broken into secret shares, and a legitimate receiver of the message should collect enough shares before he/she could reconstruct the original messages [25,26].

## 3. Proposed Framework

Our proposed framework for IoT scenarios with timing constraints is described and illustrated in this section. We have described the frequently used symbols throughout this section in Table 1. The entities of our framework comprise three roles: nodes, routers, and SDN controller. The nodes, denoted as $n_{i}$, are IoT devices. They are resources limited in carrying out complicated cryptographic operations. The routers and the SDN controller are the backbone entities of the framework, similar to any IP networks.

**Figure 2:**
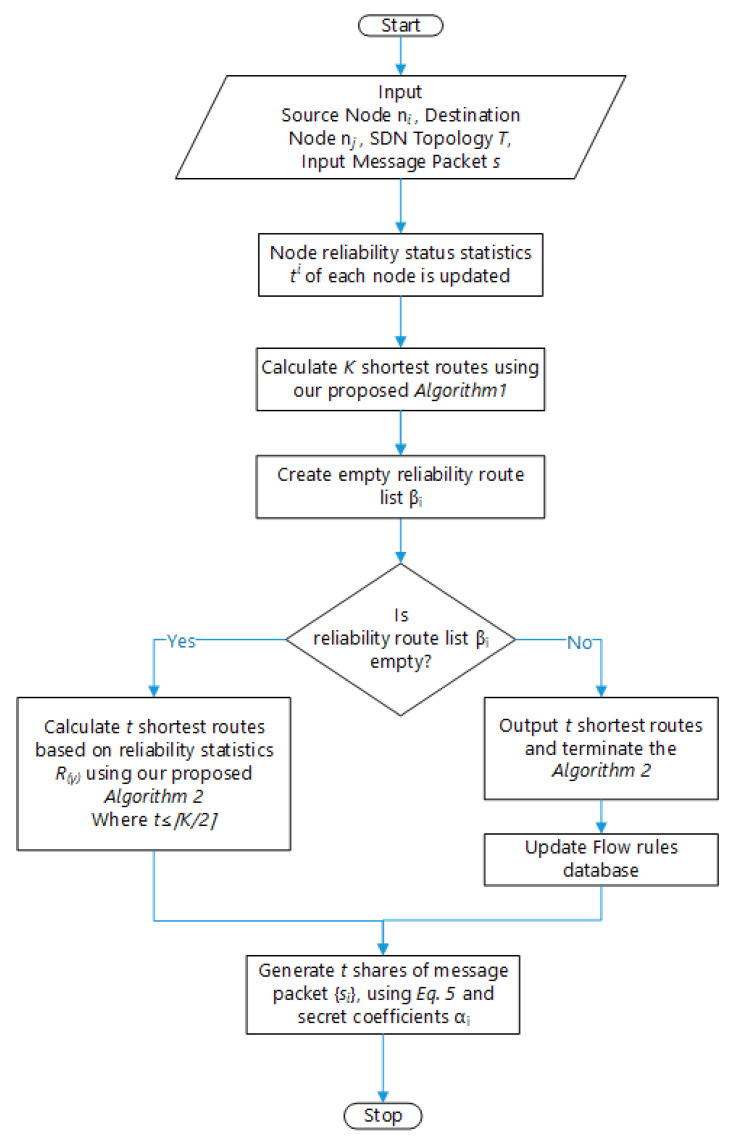
Flow chart for the mechanism of Obfuscation of routing paths in IoT-SDN.

The routers, denoted in lower-case letters as *r*, *t*, *u*, *v*, etc., are SDN-enabled and they send link states to the SDN controller, denoted as *SDN*. Therefore, the SDN controller gets the network topology and capability in terms of reliability of the switches as input, and it will compute routing paths for nodes [6]. The overall architecture of our proposed scheme is detailed in Figure 2.

**Figure 3:**
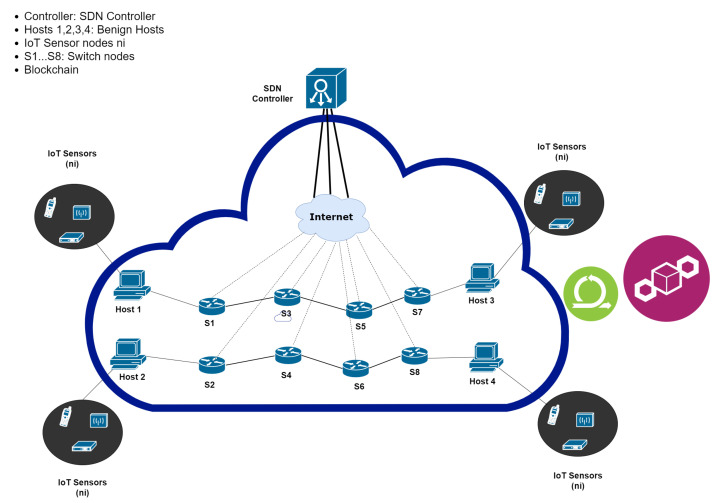
Routing path construction in IoT-SDN.

Our framework will take the reliability of the switches discussed in Section 3.1 and Section 3.2, which is determined by considering the actions of the network nodes proposed by [27]. The SDN topology shown in Figure 3 comprises of three logical and physical entities: controller, hosts, and switches.

Our first measure to protect the network traffic happens at the network layer. The SDN controller collects routing status information from the SDN-enabled routers. Then, the controller calculates multiple routes according to Algorithm 1. To obfuscate the traffic in the eye of the adversary, the SDN controller generates several routes for destination and receiver nodes in the network. It can randomly assign costs on each link so that each time the computed routes would be different. Without loss of generality, we denote any pair of communicating nodes as $n_{i}$ and $n_{j}$. The SDN controller also decides the number of routes, denoted as $\gamma_{1},\gamma_{2},\gamma_{3}$, etc., between the pair. The randomization of the routes and the number of routes all increase the difficultly for the adversary to intercept and eavesdrop the messages between nodes $n_{i}$ and $n_{j}$. The formal proof is summarized in Section 4.

**Algorithm 1:** SDN Multiple Routes Computation.

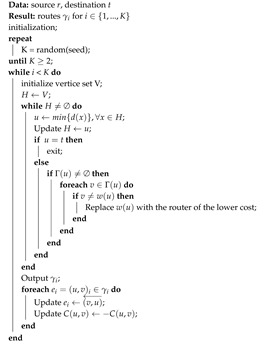



To clarify the description of the routes computation, we suppose that the source end node $n_{i}$ has its next-hop router to be *r* and the last router to the receiver end node $n_{j}$ to be *t*. Router *r* could be one of the nearby routers available to $n_{i}$. Therefore, we can view the source as *r* and the destination as *t*. The job of the SDN controller is to identify a number of routes between *r* and *t*. The proposed algorithm is built upon the shortest paths pair routing proposed in [28]. The notations are as follows, *H* is the set of potential routers from among available routes, Γ(u) is the set of neighbor routers of *u*, *K* represents routes, *r* represents source, *t* represents the destination, *u* and *v* are intermediate routers in the route from *r* to *t* and *v* is the predecessor of *u*, d(x) stands for the cost of the link to router *x*, *e* stands for the link between a pair of routers (v,u), and C(v,u) represents cost between routers *v* and *u*.

The method of calculating the routing paths are very different from the traditional networks. Because, SDN provides an upper-level vision of the network, that is the reason for controlling the network through software programming. The controller is strong enough to control the flow of packets in the network. With random assignments of link costs by the controller, each time the computed routes would be different. For this purpose, the controller must consider the capability of the network for the optimal delivery of packets across the nodes.

The routers send reliability statistics to the controller after a fixed small period. The controller will generate multiple paths based on these observations. It will output the probability of how a node will behave soon based on its performance factors.

### 3.1. Reliability of a Network Node in Terms of Probability

In order to calculate the reliability, we consider two of the three actions for a node $t_{i}^{j},j=1,\ldots ,k$, i.e., $t_{i}^{\left(1\right)}$ (Packet transfer probability by a node) and $t_{i}^{\left(2\right)}$ (the probability of a node not breaking a route) of a network node proposed by Mahmoud et al. [27]. In this study, we will introduce another factor τ (the percentage of nodes in the path) to compute the nodes’ reliability.

$t_{i}^{\left(1\right)}$: Packet transfer probabilty by a node: $n_{relay}$: count of transmitted packets in λ sessions.

(1) $$t_{i}^{\left(1\right)}=\frac{n_{relay}}{n_{total}}$$

$t_{i}^{\left(2\right)}$: The probability of node not breaking a route:

(2) $$t_{i}^{\left(2\right)}=1-\frac{n_{broken}}{\lambda }$$

$\tau_{\gamma }$: Percentage of nodes in the path: Let $n_{\gamma }$ be the total number of nodes in the path γ. Let $n_{nodes}$ be the total network nodes.

(3) $$\tau_{\gamma }=\frac{n_{\gamma }}{n_{nodes}}$$

### 3.2. Reliability of Network Path

By aggregating the reliability properties of a network nodes discussed in Section 3.1, the following equation will compute the reliability of the shortest routing path in the network. As all the actions described are independent from each other, the product of the probabilities can be used as an aggregate measure. Let $R_{\left(\gamma \right)}$ is the reliability of the path $\gamma_{i}$, i=1,2,…,k.

(4) $$R_{\left(\gamma \right)}=\left[\left\{\sum_{j=1}^{k}\left(\;\prod_{\left\{i=1,2,\ldots ,n_{\gamma }\right\}}t_{i}^{j}\right)\;\right\}+\left(1-\tau_{\gamma }\right)\right]/3$$

The reliability $R_{\left(\gamma \right)}$ is the probability of sending a packet based on the real-time parameters of the network path nodes.

### 3.3. Algorithm Based on Reliability of Network Path

After determining the number of routes *K* using Algorithm 1 as discussed above, the sender node $n_{i}$ will run a secret sharing scheme to decompose its message into *K* shares and send over the network. To demonstrate the idea, we use Shamir’s secret sharing (SSS) scheme, denoted as the (t,K) SSS explained as follows [29]. In SSS, the dealer/sender picks a secret *s* from a finite field $F_{m}$, where *m* is the field’s size. The sender then decomposes the secret *s* into *K* shares $\left\{{\left[s\right]}_{i}^{t},i=1,\ldots ,K\right\}$ such that any subset with less than the threshold t≤⌈K/2⌉ shares cannot give any knowledge to the attacker about the original secret *s*. Equations (5) and (6) describe the share generation by the sender and secret reconstruction by the legitimate receiver, respectively.

(5) $$\begin{array}{ccc} {\left[s\right]}_{i}^{t} & ≜ & \sum_{j=1}^{K-1}\alpha_{j}i^{j}+s\;\mathrm{mod}\;m, \end{array}$$

(6) $$\begin{array}{ccc} s & = & \sum_{i\in \Gamma }\gamma_{i}{\left[s\right]}_{i}^{t}\;\mathrm{mod}\;m, \end{array}$$

Once the receiver receives the secret shares, the receiver will run the reconstruction formula to see the message. If the shares arrive without tampering, the reconstructed message will be meaningful, such as control commands. However, if the adversary corrupted shares, the reconstructed message will be like garbage. Thus, the corrupted message reveals that there must be an active attacker in the routes.

We will propose our network capability based random routing algorithm, in which we use SSS to break the message into K shares and send them using the *t* routing paths generated by Algorithm 1 using the reliability framework discussed in Section 3.1 and Section 3.2.

In the proposed Algorithm 2, using the (t,K) SSS and reliability framework the sender acts as the dealer and breaks his/her secret real-time packets into shares and sends them over the network controlled by the SDN. The routes are random from time to time, and the shares follow the shortest paths determined by the SDN controller and the node capabilities to get to the legitimate receiver. No matter how computationally powerful an adversary is, it cannot break the secret message if it has the number of shares below the threshold.

**Algorithm 2:** SDN Capability-based secure Route Obfuscating Algorithm.

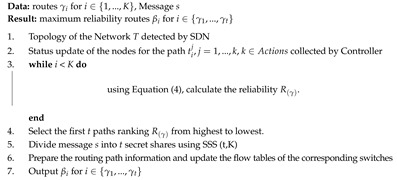



In this sense, our framework fits into post-quantum security against the quantum computer adversary. The proposed scheme does increase the path numbers in the networking part, but compared to the PKI, the message size is reduced, and also the computation time is much faster. Moreover, in our case, the security requirement of the user determines the number of shares (*t*). For high security, *t* is high near to *K*, but in case of no security, we can take *t* to be at a minimum value of 2. We will simulate the efficiency and comparison of our algorithm in Section 5.

**Figure 4:**
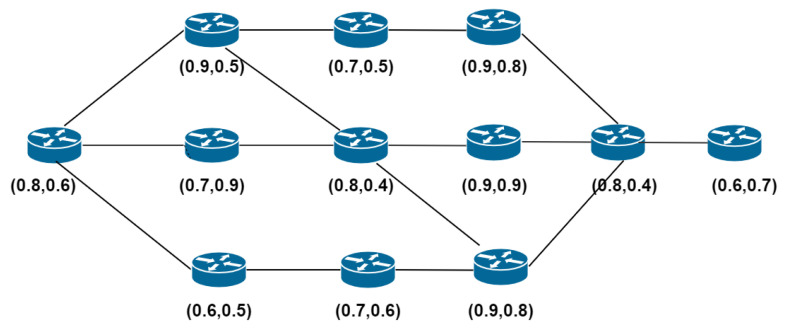
Possible routing path in IoT-SDN and node status.

Figure 4 shows the example scenario of our secret sharing-based network capability routing algorithm. Table 2 shows the computing routing paths and the reliability from source to destination. The column’s of Table 3 show the path information of a route, the second column computes the reliability in terms of reliability $R_{\left(\gamma \right)}$ of link paths. For first column S1 represents the starting node, whereas S12 represents the destination node.

After ranking the paths based on reliability values, first t paths are selected to send the information. The value of *t* can be adjusted depending upon the user requirements of security. If the security is high, the value of *t* can be taken large. Otherwise, in the case of no security requirements, *t* can be as small as permissible in the secret sharing scheme.

**Figure 5:**
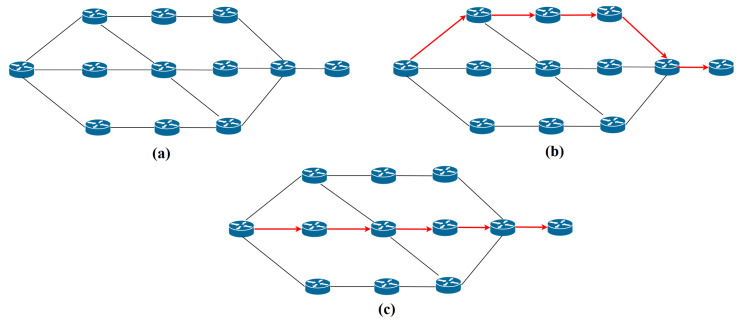
(**a**) Original Topology. (**b**) Shortest Path. (**c**) Shortest path determined using Algorithm 2 in our example scenario.

Figure 5 illustrates computed reliability status. Figure 5a shows the original topology, which contains twelve SDN-enabled switches. Figure 5b shows the topology showing the shortest path. Figure 5c shows the shortest path determined by using the capability-based routing algorithm proposed by us.

### 3.4. Secret Coefficient Exchange

There is one remaining operational step in the proposed framework: exchanging the secret coefficients between the sender and the receiver. We briefly discuss typical approaches and leave the implementation decisions to practitioners. One approach is to pre-install the secret coefficients on the IoT devices, similar to what the industry has been doing for smartphones. The basic set-up allows both parties to agree upon their secret coefficients offline as part of the system setup process. Another approach is to utilize the blockchain such as Hyperledger Fabric [30,31] as a preprocessing platform. The community of the IoT devices will maintain the blockchain. The blockchain serves as the decentralized moderator among all parties. The secret coefficients of the shares are exchanged through blockchain smart contracts where the sender and the receiver both have access. Before any communication begins, parties exchange and agree on the coefficients. Later, the parties may periodically update the secret coefficients throughout the system’s lifetime. The running of the smart contracts helps parties achieve pre-agreed information between the sender and the receiver regarding what sets of coefficients to use and at what time. Therefore, a mechanism of time synchronization is assumed in the set-up. The security of the blockchain is implied by the specific blockchain and its design, out of the paper’s scope.

## 4. Security Proof and Discussion

As the security context is related to the size of the information received by the communicating parties, therefore its perfect to use Shannon’s principle of entropy results [32]; we will present the security proof of our mechanism. If the shares are secure in transmission from source to destination, then our scheme is secure against the adversary. We have used the following definitions and properties of entropy (for details on information theory see [33]).

Let us assume the r.v.’s as ***X***, ***Y*** and ***Z***.

(7) $$H\left(X\right)=-\sum_{x\in \chi }P\left(X=x\right)\log_{2}P\left(X=x\right)$$

(8) $$H\left(X,Y\right)=-\sum_{x\in \chi }\sum_{y\in Y}P\left(X=x,Y=y\right)\log_{2}P\left(X=x,Y=y\right)$$

(9) $$H\left(X|Y\right)=\sum_{y\in Y}P\left(Y=y\right)H\left(X|Y=y\right),$$

(10) $$H\left(X|Y=y\right)=-\sum_{x\in \chi }P\left(X=x|Y=y\right)log_{2}P\left(X=x|Y=y\right)$$

To demonstrate our proof, we had used the following properties:

(11) $$\begin{array}{ccc} H(X) & \geq & H(X|Y), \end{array}$$

(12) $$\begin{array}{ccc} H(X,Y) & = & H(X)+H(Y|X), \end{array}$$

(13) $$\begin{array}{cc} = & H(Y)+H(X|Y), \end{array}$$

(14) $$\begin{array}{ccc} 0\leq & H(X) & \leq \log_{2}\chi , \end{array}$$

(15) $$\begin{array}{ccc} If\;H(Y|Z) & = & 0\;then\;H(X|Y)\geq H(X|Z), \end{array}$$

(16) $$\begin{array}{ccc} If\;H(Y|Z) & = & 0\;then\;H(X|Y,Z)=H(X|Z) \end{array}$$

In order to generate *t* secret shares (1 < *t* ≤ k) the SDN controller generates a sharing polynomial *f*∈$K_{K-1}$[Y], where K is finite field $K=F_{p}$, where *p* is prime.

(17) $$f=\alpha_{0}+\alpha_{1}Y+\ldots +\alpha_{k-1}Y^{k-1}$$

where $\alpha_{0}$ = *s* (input packet) and $\alpha_{i}\in K$(i=1,…,k−1) are selected randomly. It is assumed that no less that *t* shares, ($j_{i}\in \left[K\right],i\in t$), $f\left(j_{1}\right),\ldots ,f\left(j_{t}\right)$, of the data packet be able to decode it. The following r.v’s are considered in rest of the proof.

- *S*: Secret s
- $f_{i}$: Share f(i)(i∈[K])
- $A_{r}$: Polynomial coefficient $\alpha_{r}\left(0⩽r⩽k-1\right)$

The following are the set notations used in the proof.

- $\bar{I}$: {$j_{1},j_{2}\ldots ,j_{t}$} denotes *t* shares
- ${\bar{f}}_{k}$: {$f_{i_{1}},f_{i_{2}},\ldots ,f_{i_{k}}$} denotes k r.v. shares
- ${\bar{A}}_{k}$: {$A_{0},A_{1}\ldots ,A_{k-1}$} denotes *k* r.v. *A*
- ${\bar{A}}_{k-1}$: {$A_{1},A_{2}\ldots ,A_{k-1}$} denotes k−1 r.v. *A*

The sets $\bar{I}$ and $\bar{f_{k}}$ are bijective according to the SSS scheme.

To prove the unconditional security, we will use the proof model of Christian et al. based on entropy [34]. We need to prove the following two results.

(**1**) To prove the correctness of our scheme i.e., any *k* or more shares can be pooled together to discover the secret *s*, i.e.,

(18) $$\mathrm{for}t\geq k,\;H(S|{\bar{f}}_{t})=0.$$

(**2**) To prove that adversary did not have any information about *s*, i.e.,

(19) $$\mathrm{for}t\leq k-1,\;H\left(S|{\bar{f}}_{t}\right)=H\left(S\right).$$

### 4.1. Correctness

**Theorem** **1.** *Under the route obfuscation and secret-sharing construction, our proposed scheme is correct.*

**Proof.**  Given *k* points $\left(x_{1},y_{1}\right),\left(x_{2},y_{2}\right),\ldots ,\left(x_{k},y_{k}\right)$ of $K^{2}$, a polynomial is,

(20) $$f=\alpha_{0}+\alpha_{1}Y+\ldots +\alpha_{k-1}Y^{k-1}$$

of $K_{K-1}$[Y] ϶ $f\left(x_{i}\right)=y_{i}$ for i=1,2,…,k. The *k* coefficients $\alpha_{0},\ldots ,\alpha_{k-1}$ are determined by $\left(j_{1},f\left(j_{1}\right)\right),\ldots ,\left(j_{k},f\left(j_{k}\right)\right)$. It follows from information theory,

(21) $$H({\bar{A}}_{k}\;|\;{\bar{f}}_{k})=0$$

Furthermore, as the polynomial f and shares, uniquely calculate the coefficients $\alpha_{0},\ldots ,\alpha_{k-1}$. It follows from information theory,

(22) $$H({\bar{f}}_{k}\;|\;{\bar{A}}_{k})=0$$

using (21), (22) and (15),

(23) $$H\left(S|{\bar{A}}_{k}\right)=H\left(S|{\bar{f}}_{k}\right)$$

Also because $\alpha_{0}=s$, as illustrated before

(24) $$H\left(S|{\bar{A}}_{k}\right)=H\left(S|S,{\bar{A}}_{k-1}\right)=0.$$

Therefor, $H(S|\bar{f_{k}})=0.\mathrm{By}$ (11), the following holds

(25) $$H\left(S|{\bar{f}}_{t}\right)\leq H\left(S|{\bar{f}}_{t}\right)=0.$$

by using (14),

(26) $$H(S|\bar{f_{t}})=0.$$

This result shows the information-theoretic correctness of our route-obfuscation mechanism. ☐

**Theorem** **2.** *The proposed route obfuscation and message delivery is perfectly secure.*

**Proof.**  From (12) and (13), joint entropy of ${\bar{A}}_{k}$ and ${\bar{f}}_{0}\cup {\bar{f}}_{t}$ is

(27) $$\begin{array}{c} H({\bar{A}}_{k},{\bar{f}}_{0}\cup {\bar{f}}_{k-1})=H(\bar{f_{0}}\cup {\bar{f}}_{k-1})+H(\bar{A_{k}}\;\left|\;\bar{f_{0}}\cup {\bar{f}}_{k-1}\right) \end{array}$$

from (21),

(28) $$=H({\bar{f}}_{0}\cup {\bar{f}}_{k-1}).$$

Similarly, from (12) and (13),

(29) $$\begin{array}{c} H\left({\bar{A}}_{k},{\bar{f}}_{0}\cup {\bar{f}}_{k-1}\right)=H({\bar{A}}_{k})+H({\bar{f}}_{0}\cup {\bar{f}}_{k-1}\;\left|\;{\bar{A}}_{k}\right) \end{array}$$

from (22),

(30) $$=H({\bar{A}}_{k}).$$

Hence,

(31) $$H({\bar{f}}_{0}\cup {\bar{f}}_{k-1})=H({\bar{A}}_{k}),$$

as $\bar{f_{0}}=\alpha_{0}=s$,

(32) $$\begin{array}{c} H(S,{\bar{f}}_{k-1})=H(S,{\bar{A}}_{k-1}) \end{array}$$

Using (12), (13), Equation (32) can be written as

(33) $$H\left(S\right)+H(\bar{f_{0}}\cup {\bar{f}}_{k-1}\left|S\right)=H({\bar{A}}_{k-1})+H\left(S|{\bar{A}}_{k-1}\right)$$

The coefficients $\alpha_{0},\ldots ,\alpha_{k-1}$ are chosen randomly in K. As a result,

(34) $$H({\bar{A}}_{k-1})=\log_{2}{\left|K\right|}^{k-1}.$$

As coefficients are independent from the secret s, i.e., H (${\bar{A}}_{k-1}$) = H(*S*), therefore

$$H({\bar{f}}_{k-1}|S)=\log_{2}{\left|K\right|}^{k-1}.$$

using (11),

(35) $$H\left({\bar{f}}_{k-1}\right)\geq H\left({\bar{f}}_{k-1}|S\right),$$

(36) $$H\left({\bar{f}}_{k-1}\right)\geq \log_{2}{\left|K\right|}^{k-1}$$

using (14),

(37) $$H\left({\bar{f}}_{k-1}\right)\leq \log_{2}{\left|K\right|}^{k-1},$$

therefore,

(38) $$H\left({\bar{f}}_{k-1}\right)=\log_{2}{\left|K\right|}^{k-1},$$

from (12) and (13), we can write Equation (32) as

(39) $$\begin{array}{c} H\left({\bar{f}}_{k-1}\right)+H(S|{\bar{f}}_{k-1})=H\left({\bar{A}}_{k-1}\right)+H\left(S\right|{\bar{A}}_{k-1}) \end{array}$$

As

(40) $$H\left({\bar{f}}_{k-1}\right)=H{\bar{A}}_{k-1})=\log_{2}{\left|K\right|}^{k-1}$$

(41) $$H(S|{\bar{A}}_{k-1})=H\left(S\right),$$

hence,

(42) $$H(S|{\bar{f}}_{k-1})=H\left(S\right).$$

Using (11)

(43) $$\begin{array}{ccc} H(S) & \geq & H(S|{\bar{f}}_{t}) \end{array}$$

(44) $$\begin{array}{cc} \geq & H(S|{\bar{f}}_{k-1}) \end{array}$$

(45) $$\begin{array}{cc} = & H(S). \end{array}$$

This implies,

(46) $$H(S|{\bar{f}}_{t})=H\left(S\right).$$

This proves that our route obfuscation and message delivery scheme is perfectly secure in information-theoretic sense. ☐

### 4.2. Discussion

In this section briefly discuss the possible mitigation of the attacks using our scheme.

- Our scheme is a plausible way to guard against MITM attacks. In southbound communication, Transport Layer Security (TLS) is used to protect the network from malicious hosts. Open Flow suggests secure southbound communication through TLS. Systems that do not use TLS are prone to MITM attacks. Many types of MITM attacks exist in the literature. Eavesdropping and packet replaying are some of the kind of MITM attacks. This is the most common attack on privacy. Attackers can easily discover the content of the interaction by snooping the data. To avoid the latency of using TLS support, we use the SSS scheme. Our scheme divides the information into shares, which makes it impossible for adversary to get any use full information out of the packet. If any malicious host replays the packets, the destination node will not be able to decode and result in dropping the fake malicious packet. Moreover, the destination node can predict the presence of an intruder in the network path.
- Our scheme can also help detect the adversarial or fake nodes in the network by analyzing the data. Packets are replayed or sent by nodes that do not allow the destination node to decode the packets, thereby identifying node disrupting network and further being blocked.

## 5. Simulations

This section, analyses the result of our proposed scheme. We prototyped our methodology and the proposed mechanism, and its usefulness and execution cost is assessed in this section. To emulate our network, We adopt Mininet [35] (version 2.3.0), an open source environment, which is prominently utilized for imitating OpenFlow network environments. We generated different network topologies listed in Table 4. One of the topologies has 12 nodes, see Figure 5. We simulated the network environment in python on Ubuntu 18.04 LTS, using our workstation with configuration (Intel(R) Core TM i7-4500U CPU @ 1.80 GHz 2.40 GHz). For our scheme, Ryu [36] (version 4.24), an open source, free SDN controller supporting python language, is selected. The virtual switch use Open vSwitch (OvS) [37](version 2.10.0). Open vSwitch, often abbreviated as OVS, is an implementation requires virtual environments. The purpose of OVS is providing a switching stack for hardware virtualization environments at the same time as supporting more than one protocols and requirements utilized in networks. Table 5 summarizes the software that was used to implement our proposed mechanism.

We added two modules by extending the Ryu controller: (i): Evaluating the nodes using the capability mechanism. (ii): Security level according to the real-time security requirement of the receiver.

We will evaluate our secret route-obfuscation algorithm’s performance by calculating the overhead introduced compared to the shortest path algorithm and the time complexity. To evaluate the overhead introduced while splitting the message into shares, as illustrated in Algorithm 2, we compare the results with the shortest path algorithm using Algorithm 1 in our implementation.

### 5.1. t Paths Using Algorithm 2

**Figure 6:**
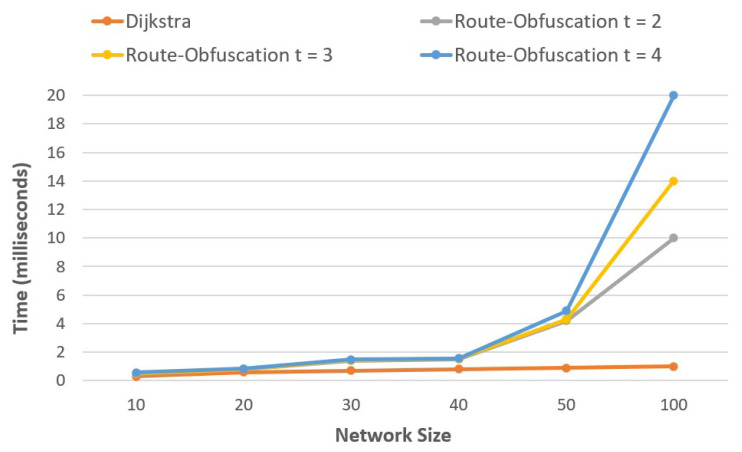
Time complexity with different network nodes.

Figure 6 illustrates the overhead of our security mechanism over the shortest path. In this study, the network performance is examined according to the number of routers. However, the positions of the hosts and routers are kept constant. We simulated K-shortest path using Algorithm 1 and then choose *t* paths using Algorithm 2, where t≤⌈K/2⌉. To probe the effect of the size of network, we consider SDN IoT networks with 10, 20, 30, 40, 50, and 100 nodes. The *X*-axis represents the network size and *Y*-axis represents time in milliseconds to construct *K* paths and selecting best *t* paths. Results indicate that the efficiency of calculating and choosing the paths is acceptable when *t* is small.

Table 4 shows the network configuration. For each network, we varied the number of routers and calculated the links. The average minimum and the maximum of nodes in the shortest paths are determined.

We replicated our experiments 20 times and calculated the average time complexity for constructing the possible routing paths, as the network size affects the time. When the first transfer starts, the controller takes some time to discover the network’s topology and update the flow tables. This time is calculated as an average of 6.3 s. Of course, this time depends on the specification of the computer used. We have removed this time as it is the delay, which could affect the network performance time.

**Figure 7:**
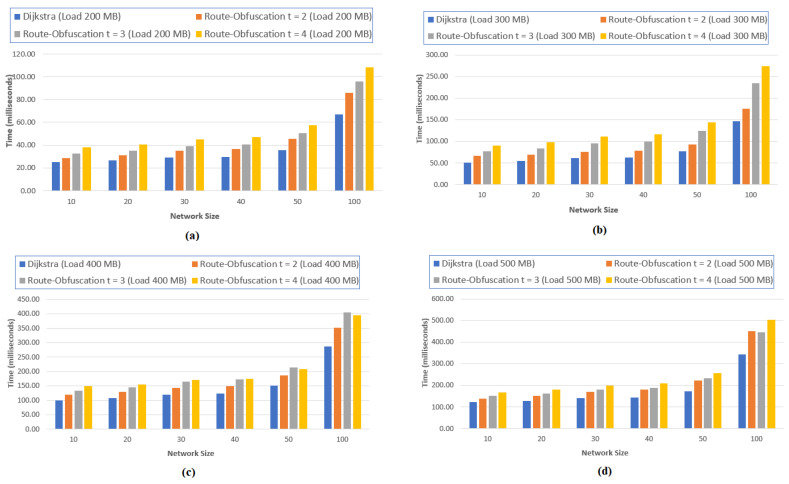
Average execution time of our route obfuscation algorithm. (**a**) Load = 200 MB; (**b**) Load = 300 MB; (**c**) Load = 400 MB; (**d**) Load = 500 MB.

Simulation tests were carried out by taking variable message lengths of 200, 300, 400, and 500 megabytes and shares of the input packet of size 3, 4, and 5 per host pair. While the number of routers is increased, the hosts and IoT nodes’ positions are kept the same. Figure 7 (a-d) shows the average time of our implementation. In the graph, network size is plotted on *X*-axis and the average time in milliseconds for our obfuscation route algorithm to calculate the routing paths on *Y*-axis. The graph shows that the execution time of the algorithm increases linearly with increase in network size. Dijkstra’s execution time is the lowest, as it is the most basic algorithm used here for comparison. because of more than one path, our algorithm’s execution time shows that it is an acceptable trade-off between security and efficiency.

**Figure 8:**
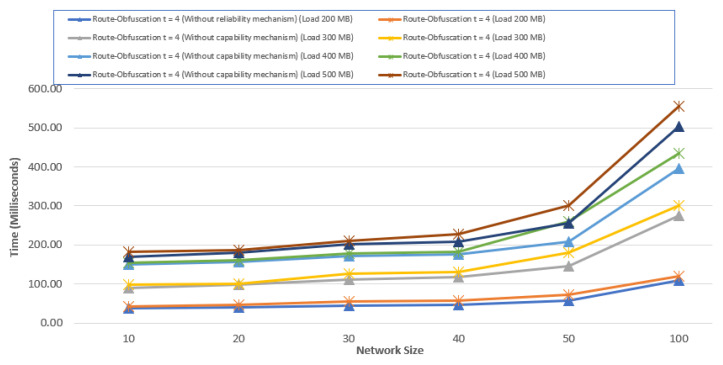
Average execution time comparison of using the route obfuscation algorithm for t=4.

We compared the running time of our algorithm before and after configuring the reliability mechanism to determine the paths. In the graph, network size i.e., nodes is plotted on the *X*-axis and the average time in milliseconds on *Y*-axis by taking message lengths of 200, 300, 400, and 500 MB. Average results are obtained after replicating experiments 20 times each. The results of Figure 8 indicate that the reliability mechanism has chosen the paths efficiently as compared to the one without. Therefore, the feasibility of incorporating the security mechanism can be considered where speed can be compromised over security. As we are choosing the shortest path in our route obfuscation approach, the increase in time is approximately linear.

### 5.2. Time Complexity

Let us consider formal definition for our IoT SDN Network. For dijkstra algorithm the running time complexity is O(m+nlogn), and for our t-shortest path route-obfuscation algorithm is O(t(m+nlogn)), where *m* and *n* represents nodes and edges respectively.

## 6. Conclusions

With the threat of upcoming quantum adversaries, cryptography schemes built upon PKI that rely on number-theoretic hard problems are vulnerable. Besides, the limited computing resources and energy storage in the IoT devices make the efficiency of using PKI to establish critical message passing in the scale of milliseconds unrealistic. To overcome both challenges, we propose to use information-theoretic secret sharing with route-obfuscation to provide security against certain adversaries in realistic settings. Our construction achieves information-theoretic security in message encryption and increases the difficulty for adversaries to intercept messages or modify the messages. Our scheme comes with theoretic proofs and simulation validation. The proposed scheme is also efficient as it has a time complexity of a few milliseconds, validated by the simulation results. In the future, we will investigate the relationship between the number of random routes and time complexity, as well as countermeasures for active adversaries on the routes after the receiver node detects the failure of reconstructed messages.

## Figures and Tables

**Table 1:** Commonly used symbols.

Symbol	Description
S1…S12	Routers
$n_{i}$	IoT nodes
*T*	SDN Topology
*s*	Input Message Packet
$t^{i}$	Node reliability status
$\beta_{i}$	Reliability Path List
$\tau_{\gamma }$	Percentage of nodes in the path
$t^{1}$	Packet transfer probability by a node
$t^{2}$	Probability of a node not breaking a route
*K*	Total number of paths
$\gamma_{i}$	Route
*d*	Cost of the link
*e*	Link between a pair of router
*C*	Cost between a pair of routers
*V*	Set of nodes in the Topology
Γ	Set of neighbour routers
*t*	Number of shortest routes/shares
SSS	Shamir Secret Sharing
$\alpha_{i}$	Secret Coefficients
λ	Session count
$n_{total}$	Packet count in λ sessions
$n_{broken}$	Broken session count in last λ sessions
$n_{\gamma }$	Node count in the path γ
$n_{nodes}$	Node count in the Network
*H*	Set of potential routers from among available routes
$R_{\gamma }$	Reliability of the path $\gamma_{i}$

**Table 2:** Routing path reliability.

Route	Path	Reliability
$\gamma_{1}$	S1−>S2−>S6>S7−>S8−>S12	0.2617
$\gamma_{2}$	S1−>S5−>S6>S11−>S8−>S12	0.2473
$\gamma_{3}$	S1−>S2−>S3>S4−>S8−>S12	0.2527
$\gamma_{4}$	S1−>S9−>S10>S11−>S8−>S12	0.2285
$\gamma_{5}$	S1−>S2−>S6>S11−>S8−>S12	0.2604
$\gamma_{6}$	S1−>S9−>S10>S11−>S6−>S7−>S8−>S12	0.1508
$\gamma_{7}$	S1−>S5−>S6>S2−>S3−>S4−>S8−>S12	0.1566
$\gamma_{8}$	S1−>S5−>S6>S7−>S8−>S12	0.2493

**Table 3:** The updates status of nodes in the topology.

Nodes	$t^{\left(1\right)}$	$t^{\left(2\right)}$
S1	0.8	0.6
S2	0.9	0.5
S3	0.7	0.5
S4	0.9	0.8
S5	0.7	0.9
S6	0.8	0.4
S7	0.9	0.9
S8	0.8	0.4
S9	0.6	0.5
S10	0.7	0.6
S11	0.9	0.8
S12	0.6	0.7

**Table 4:** Network configuration.

Network	No. of Nodes	Number of links	Avg. Min. Nodes	Avg. Max. Nodes
			in a path	in a path
Network1	10	15	4	7
Network2	20	28	7	13
Network3	30	39	6	12
Network4	40	53	8	19
Network5	50	71	5	21
Network6	100	112	7	27

**Table 5:** Experimental and evaluation software.

Software Application	Operation	Version release
Mininet	Emulator for Network	2.3.0
Ryu	SDN Controller	4.24
OpenFlow	Communications Protocol	1.3
Linux	Operating System	Ubuntu 18.04 64bit
VM VirtualBox Oracle	Virtualizer machine	5.2.4
